# Anatomical study of the female reproductive system and bacteriome of *Diaphorina citri* Kuwayama, (Insecta: Hemiptera, Liviidae) using micro-computed tomography

**DOI:** 10.1038/s41598-020-64132-y

**Published:** 2020-04-28

**Authors:** Ignacio Alba-Alejandre, Javier Alba-Tercedor, Wayne B. Hunter

**Affiliations:** 1 Department of Zoology, Faculty of Sciences, University of Granada, Campus de Fuentenueva, Granada, Spain; 2 U.S. Department Agriculture, Agricultural Research Service, Fort Pierce, Florida USA

**Keywords:** 3-D reconstruction, X-ray tomography, Imaging, Entomology, Biological techniques, Zoology

## Abstract

Huanglongbing (HLB) (citrus greening disease) is one of the most serious bacterial diseases of citrus. It is caused by (1) *Candidatus* Liberibacter africanus, transmitted by *Trioza erytreae* and (2) *C*.L. asiaticus and *C*.L. americanus, transmitted by *Diaphorina citri*. As part of a multidisciplinary project on *D. citri* (www.citrusgreening.org), we made a detailed study, using micro-computed tomography, of the female abdominal terminalia, reproductive system (ovaries, accessory glands, spermatheca, colleterial (= cement) gland, connecting ducts, and ovipositor) and bacteriome, which we present here. New terms and structures are introduced and described, particularly concerning the spermatheca, ovipositor and bacteriome. The quality of images and bacteriome reconstructions are comparable, or clearer, than those previously published using a synchrotron or fluorescence *in situ* hybridisation (FISH). This study: reviews knowledge of the female reproductive system and bacteriome organ in *D. citri*; represents the first detailed morphological study of *D. citri* to use micro-CT; and extensively revises existing morphological information relevant to psylloids, hemipterans and insects in general. High quality images and supplementary videos represent a significant advance in knowledge of psylloid anatomy and are useful tools for future research and as educational aids.

## Introduction

Huanglongbing (HLB) or citrus greening disease is considered to be the most serious disease threatening the citrus industry^1^. HLB is a bacterial disease caused by *Candidatus* Liberibacter spp. It causes fruit loss as well as the production of small, bitter, unpalatable fruit, and eventually tree death. To date, HLB has spread to over 40 countries in Asia, Oceania, North America and South America^2,3^. There are two species of psylloids known to be vectors of the disease^4–6^: (1) *Trioza erytreae* (Del Guercio) (Hemiptera: Triozidae) which vectors *C*. L. africanus, and (2) *Diaphorina citri, Kuwayama* (Hemiptera: Liviidae) which vectors *C*. L. asiaticus, and *C*. L. americanus [https://www.cabi.org/isc/datasheet/16565].

Since *D. citri* was first discovered in Taiwan in 1907^7^, it has become the major vector of HLB in Asia and USA. This psylloid predominantly acquires the bacterium during nymphal feeding on infected citrus plants^8^. When *D. citri* is infected with *C*. Liberibacter its fecundity increases and it produces more offspring^9^.

A number of studies have described the female anatomy of psylloid species^10–23^. However, few studies have focused on the female reproductive system of *D. citri*^24,25^. Those studies that have been done, have used dissected specimens and descriptions based on light and electron microscopy photographs with some schematic drawings.

Many insects, and in particular different groups of sap-sucking hemipterans, have endosymbiotic microorganisms located within a specialized organ, the bacteriome (also known as the mycetome). While the microorganisms contained within the bacteriome seem to primarily provide essential amino acids and vitamins, they also have a defensive role. Within the bacteriome there are bacteriocytes which are specialized cells that provide nutrients and shelter for these microorganisms. The bacteriome is clearly visible in juvenile stages but it reduces in size or disappears in mature individuals, once the microorganisms have passed into the eggs (e.g.^26–33^). Several studies on the bacteriome of *D. citri* have been made and include: visualization of the bacteriome in nymphs^34^ and adults^35^ using fluorescence microscopy; the behaviour of symbionts during transovarial transmission and development^35^; localization and dynamics of *Wolbachia* microorganisms in nymphs and eggs^36^; and inter-population variability in endosymbiont densities^28^.

Traditional insect-dissection techniques were first used more than 400 years ago by Aldrovandi^37^ and Malpighi^38^. While this technique is useful and has resulted in thousands of papers on the internal anatomy of insects, it has limitations because it distorts the spatial arrangement of internal structures. A relatively recent technique, known as micro-computed tomography (micro-CT), which is based on X-rays, allows visualization of the internal anatomy *in situ*, without the need for dissection; results have been validated by comparing them with classical destructive methodologies^39,40^. Thus, micro-CT has been used to elucidate various aspects of insect anatomy (e.g.^39–77^) and behavioural/anatomical adaptations (e.g.^78–82^). Synchrotron X-ray tomography has been used to elucidate the general 3D configuration of the bacteriome of *Orosius albicinctus* (Hemiptera: Cicadellidae)^33^, and micro-CT has been used to locate and study the mycangia (cuticular cavities where symbiotic fungi proliferate^26,83^) of ambrosia beetles (Coleoptera: Curculionidae: Scolytinae)^65,73^.

Use of micro-CT facilitated a very detailed study of the male reproductive system of *D. citri* that included a revision of the male reproductive system of psylloids in general^84,85^. In this context, the main aim of the current study was to use micro-CT to extend these anatomical studies to the female reproductive system of *D. citri*. We present here the extensive use of micro-CT techniques to reveal, in detail, the anatomy of the female reproductive system (terminalia, ovaries, accessory glands, spermatheca, colleterial/cement gland, connecting ducts and ovipositor), and the bacteriome organ. This improvement on current knowledge helps us understand the morphology and functional anatomy of structures in their natural anatomical position, avoiding deformations that typically occur using standard dissection and/or slide preparation techniques. We also present videos as supplementary information to provide an accurate view of the actual position and internal components of the organs and structures. Spinning animations, using different rotational axes, permit exceptional views of the minutiae of structures from different 3D perspectives (Supplementary Videos S1–S3). These are useful tools for future research and as teaching aids (see also www.citrusgreening.org).

## Results

**Figure 1:**
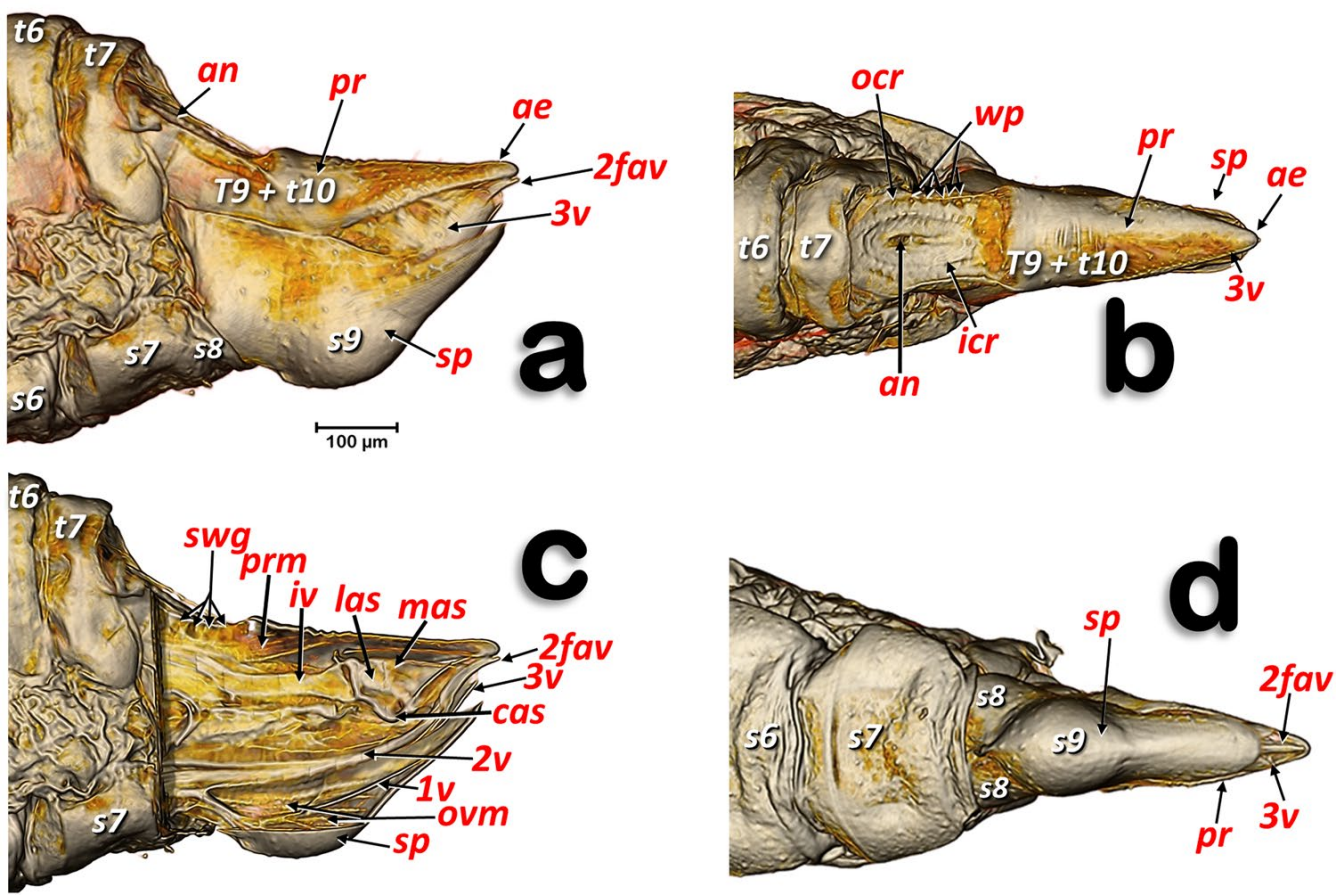
Volume-rendered images of female terminalia in different views. Left-lateral (**a**, **c**), dorsal (**b**), ventral (**d**) and medial-left virtual box-cut view (**c**). Abbreviations: 2fav = fused apical part of the 2^nd^ valvulae; ae = apical extension: an = anus; cas = centro-apical sclerite; icr = inner circum-anal ring; iv = intervalvular sclerite; las = latero-apical sclerite; mas = medial apical sclerite; ocr = outer circum-anal ring; ovm = ovipositor protractor musculature; pr = proctiger; prm = proctodeal musculature; sp = sub-genital plate; swg = sub-cuticular wax glands; wp = wax pores. Abdominal tergites and sternites are labelled sequentially with the letter ‘t’ and ‘s’, respectively.

The structures of the external female terminalia are shown in Fig. 1. Visible are a dorsal proctiger (a narrow dorsal dome-shaped plate) with a pointed apical extension (Fig. 1a,b). The anus opens dorsally in the basal part of the proctiger, at the end of a longitudinal central depression, and is encircled by double concentric (outer and inner) circum-anal rings. Wax pores are visible in the outer circum-anal ring (Fig. 1b). Ventrally appears: the subgenital plate (with a slightly bi-lobed raised appearance, narrowing and apically rounded); the lateral margins of the proctiger; and some apical parts of the ovipositor (2^nd^ and 3^rd^ valvulae) (Fig. 1d). Laterally, the proctiger and the subgenital plate (with a narrow spoon-shaped appearance) are conspicuous, and are visible between both the apical parts of 2^nd^ and 3^rd^ valvulae of the ovipositor (Fig. 1a). A small latero-apical notch appears on each 3^rd^ valvula (Fig. 7j).

**Figure 2:**
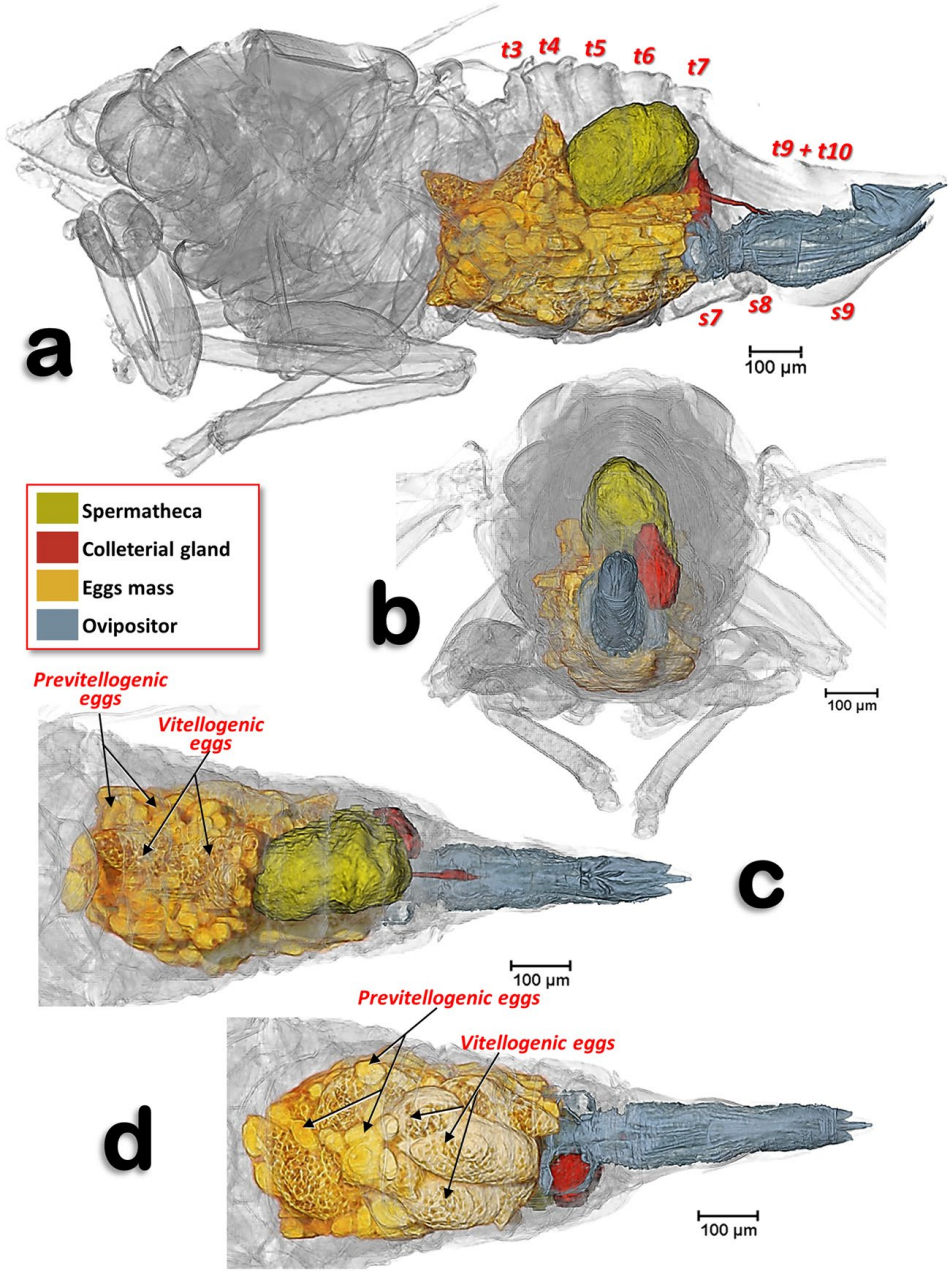
Volume-rendered images of an older mature adult female showing the reproductive system in its anatomical position, in different views. Left-lateral (**a**), posterior (**b**), dorsal (**c**) and ventral (**d**). Abdominal tergites and sternites are labelled sequentially with the letter ‘t’ and ‘s’, respectively. Note that neither the intervalvular basal sclerite nor the latero-basal plate sclerites of the ovipositor are rendered.

**Figure 3:**
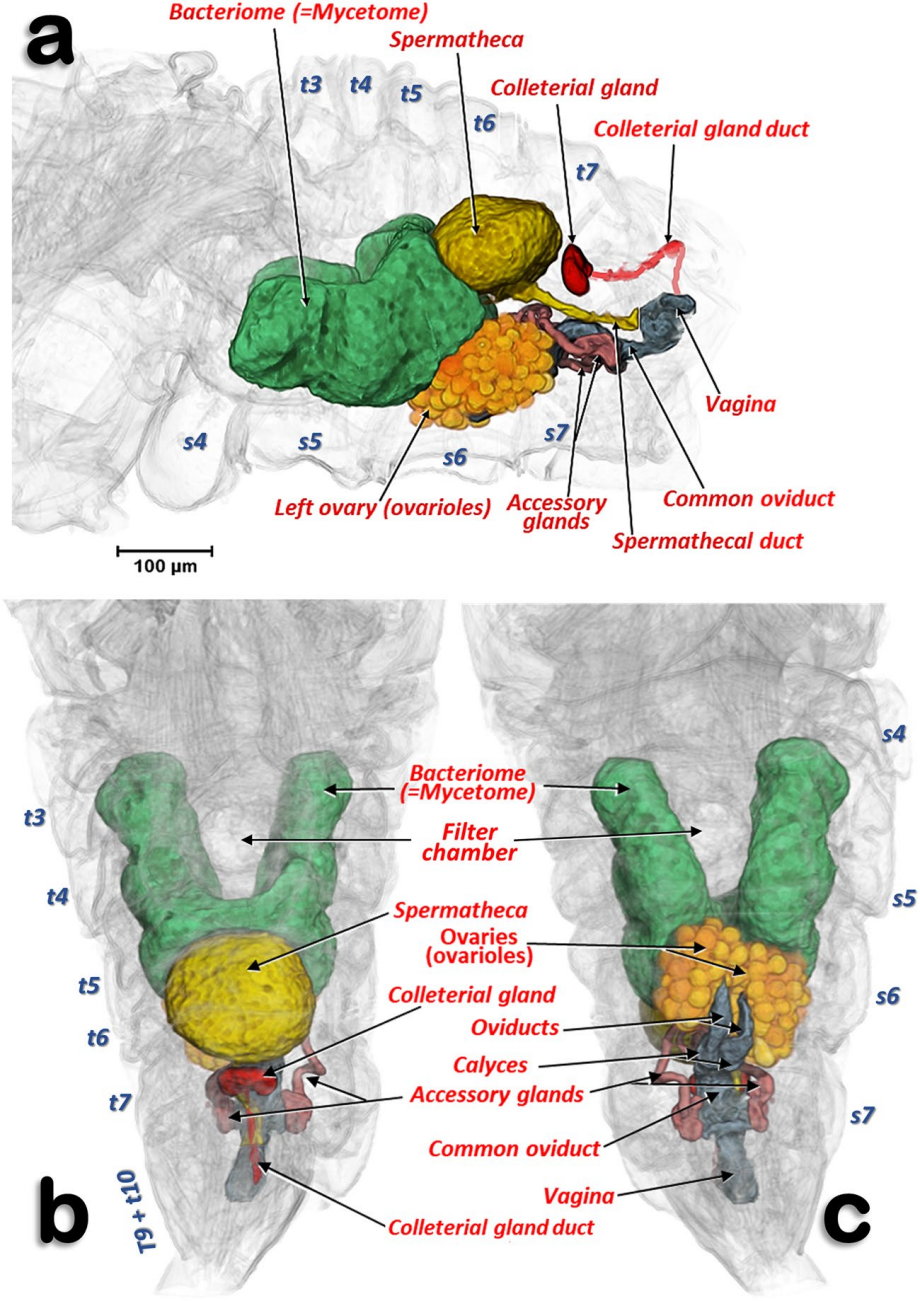
Volume-rendered images of an adult young female showing the reproductive system organs, bacteriome and the filter chamber, in its anatomical position. Left-lateral (**a**), dorsal (**b**) and ventral (**c**). Note that the ovipositor is not rendered.

**Figure 4:**
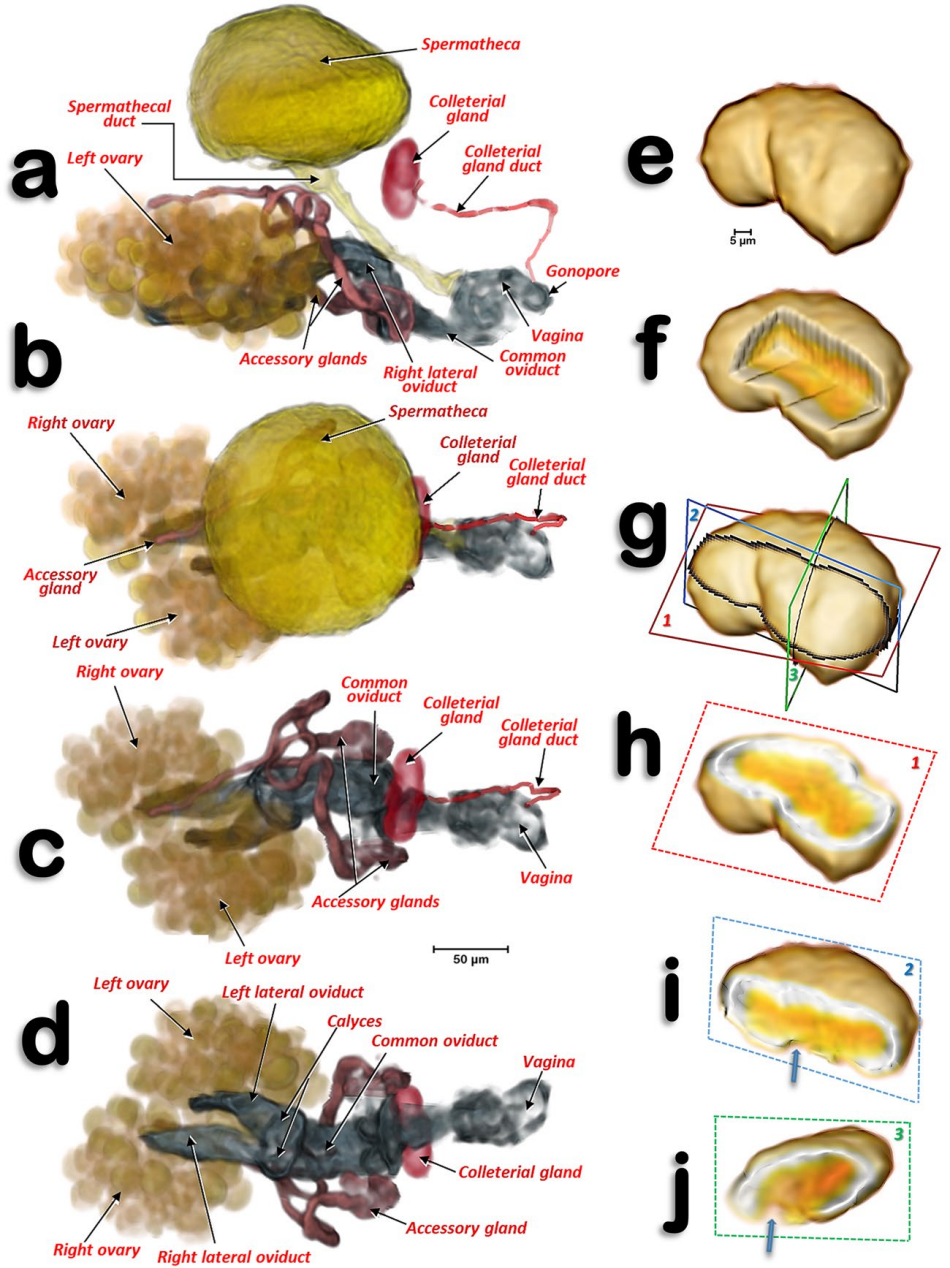
Volume-rendered images of the reproductive system and organs of a young adult female in different views. Left-lateral (**a**), dorsal (**b**,**c**), and ventral (**d**). For clarity, the spermatheca is not rendered in (**c**,**d)**. Colleterial gland in left antero-dorsal view (**e**–**i**) and different views of the internal structure after virtual cuts: box-cut (**f**) and different plane cuts (**h**–**j**), as defined in (**g)**. In (**i**,**j)** the blue arrows point to the connection of the colleterial gland duct.

**Figure 5:**
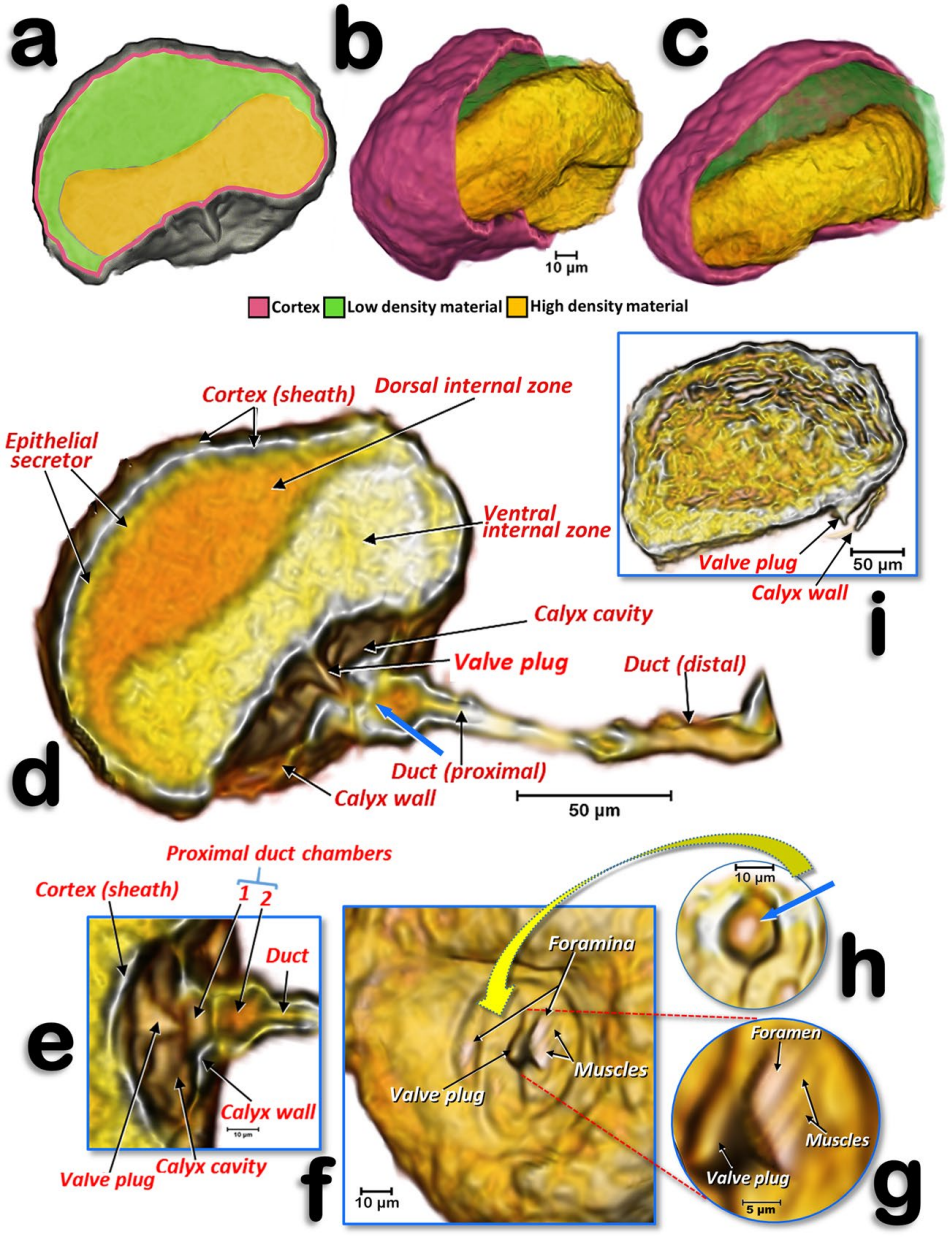
Volume-rendered images of the spermatheca of a young adult female (**a**–**h**) and of an older adult female, where the calyx is not rendered (**i**). Left-lateral section (**a**,**d**,**e**), details of the structures inside the calyx and proximal spermathecal duct (**e–h**), internal view proximal at the level where the calyx connects (**f**), a detailed view showing the bigger foramen and the closing muscles (**g**), and pierced wall that separates the proximal duct chamber from the interior of the calyx - the blue arrow points to the hole where the valve plug inserts (**h**).

**Figure 6:**
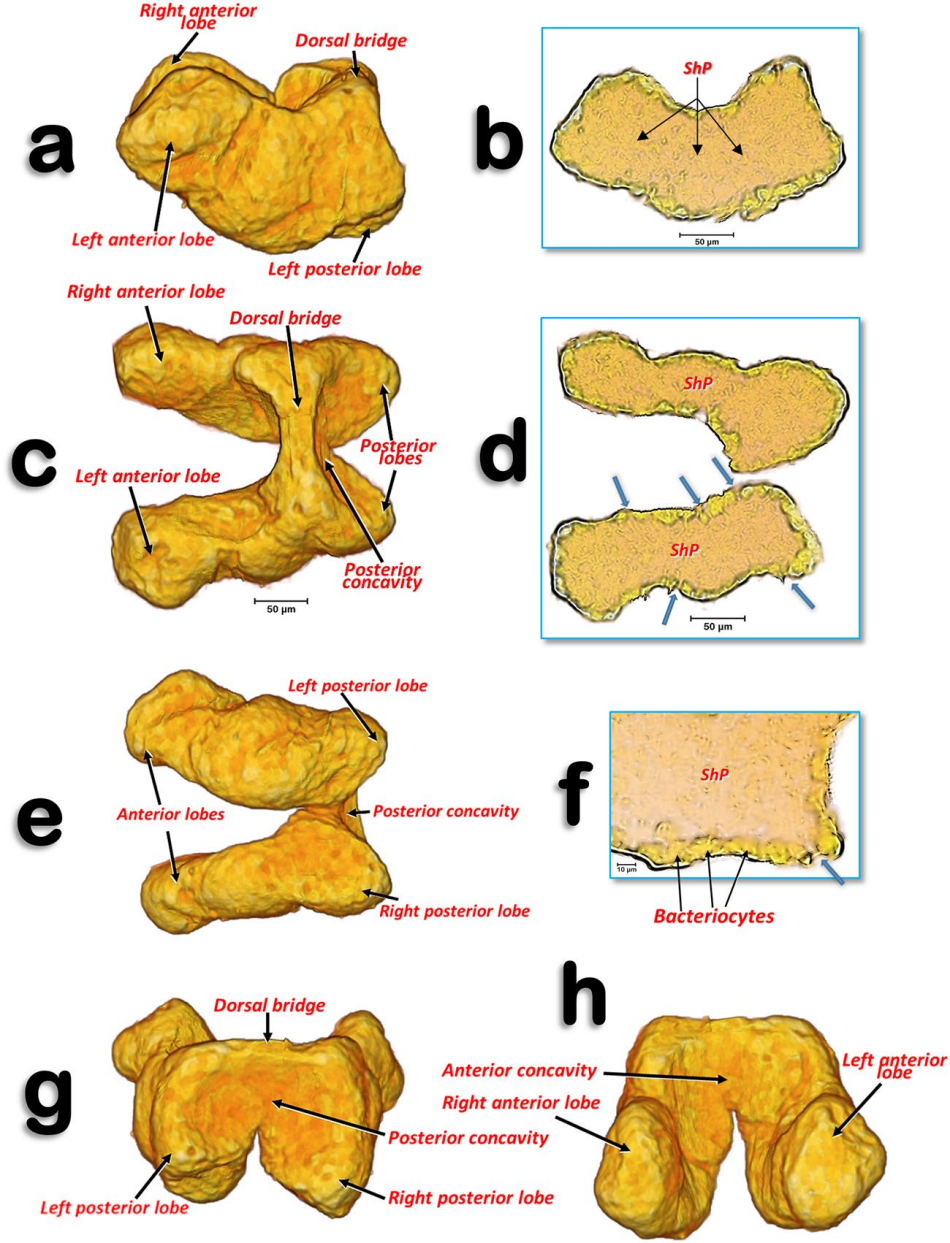
Volume-rendered images of the bacteriome of a young adult female in different perspective views. Left-lateral (**a**), dorsal (**c**), ventral (**e**), posterior (**g**) and anterior (**h**). Slice sections: left-lateral (**b**), dorsal (**d**) and details of a left-lateral section (**f**). The blue arrows indicate the places where the syncytium reaches the surface of the bacteriome. Abbreviation: ShP = syncytium harbouring *Profftella*.

**Figure 7:**
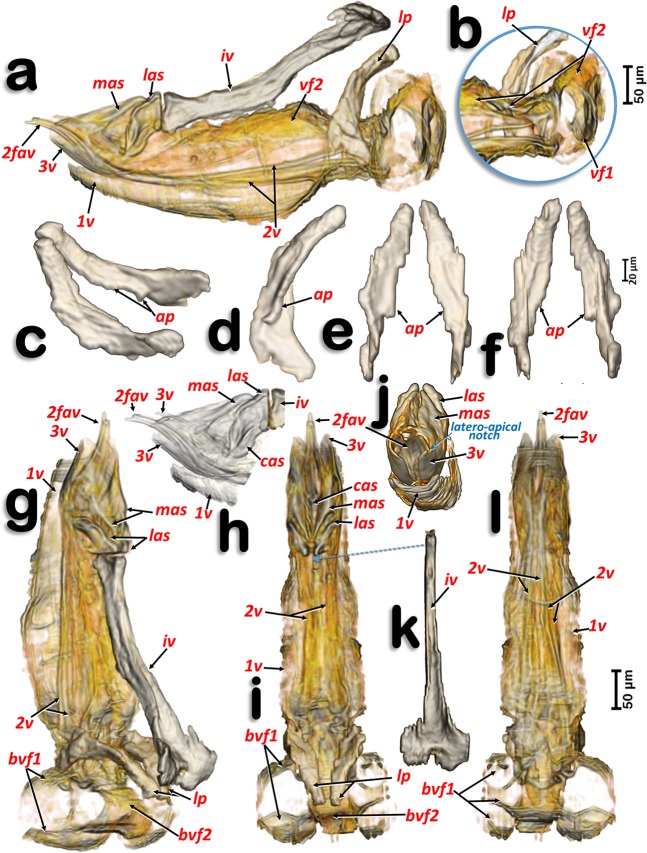
Volume-rendered images of the ovipositor in different perspective views. Right-lateral (**a**,**b**,**h**), right dorso-lateral (**g**), dorsal (**i**), posterior (**j**) and ventral (**l**). Latero-basal plate sclerites in different views: right dorso-lateral (**c**), internal side of the left sclerite (**d**), posterior (**e**) and frontal (**f**). Detail of a right apical view, using software to show the apical sclerites and valvulae (**h**). Dorsal view of the intervalvular basal sclerite (**k**). Abbreviations: 1 v, 2 v and 3 v = 1^st^, 2^nd^ and 3^rd^ valvulae; 2fav = fused apical part of the 2^nd^ valvula; ap = latero-basal plate internal apodeme; cas = centro-apical sclerite; iv = basal intervalvular sclerite; las = latero-apical sclerite; lp = latero-basal plate sclerite; mas = medio-apical sclerite; vf1 and vf2 = 1^st^ and 2^nd^ valvifera.

In the specimens studied, the abdominal volume occupied by the female reproductive system increases with maturity and with egg development. Thus, while in the young adult female, it occupies less than ¼ of the abdomen (Fig. 3; Supplementary Video S1), in older adult females (with developed vitellogenic eggs) the abdominal cavity appears to be completely occupied by the reproductive organs, and particularly by the eggs (Fig. 2).

**Figure 8:**
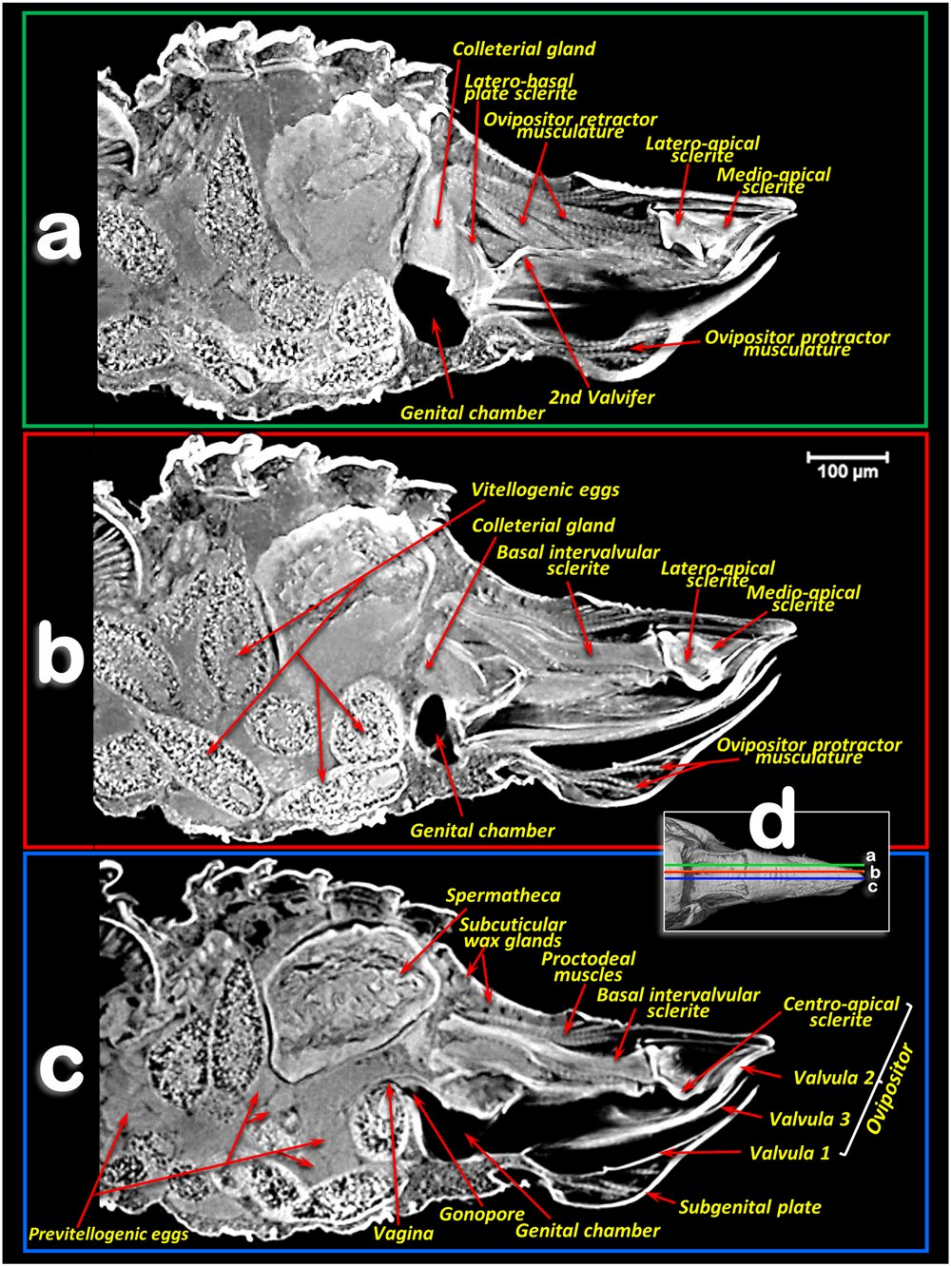
Amira´s multiplanar sagital section slices of an older female adult (**a**–**c**), according to plane cuts shown in **d** (slices **a**,**b**: 23 μm thick and slice **c**: 3 μm thick).

The reproductive organs are at a medio-ventral position in the apical third of the abdominal cavity. This is evident in the young adult female (Fig. 3; Supplementary Video S1), but not in older adult females where the abdomen is full of eggs (Fig. 2). There are two ovaries, each containing ca. 40–50 ovarioles (we counted 40 in the right and 46 in the left) in a configuration similar to a blackberry fruit (Fig. 2a,c). The ovarioles of each ovary join in a lateral oviduct that enlarges distally in a calyx. Right and left oviducts come together forming a common oviduct, into which a tubular accessory gland flows on each side, and distally enlarges to form the vagina (Figs. 3, 4a–d; Supplementary Video S1). The vagina ends in a gonopore that opens to the genital chamber at the base of the ovipositor (Fig. 8c).

The spermatheca is situated dorsal to the ovaries and in a central position; it is spherical in shape and its surface has small semi-spherical protuberances which give it a rough appearance. The spermatheca is connected to the vagina by a spermathecal duct (Figs. 3a,b, 4a,b). The colleterial (= cement) gland appears situated in a medial position, dorsal to the common oviduct, and posterior to the spermatheca (Figs. 2, 3, 4a–d; Supplementary Video S1). It has a denser external layer (appearing close to white in colour) with less dense internal contents (appearing yellowish in colour) (Fig. 4h–j). From the central posterior side, the colleterial gland is connected to the colleterial gland duct (Fig. 4i–j), through which it releases gland secretions into the space between the valvulae of the ovipositor (Fig. 2a,c).

The structure of the spermatheca is rather complex (Fig. 5; Supplementary Video S2). The main body of it is spherical/globular and it has an external cortex (sheath) that surrounds an epithelial secretor layer. Internally, it presents as two cavity zones: a dorsal internal zone (less dense in X-ray) and a ventral internal zone (denser in X-ray); these appear as green/yellow structures (Fig. 5a-c) and orange/yellow structures (Fig. 5d), respectively. In older adult females, the spermatheca appears laterally compressed (Fig. 2b,c) and internally lacking the two separate zones (Figs. 5i and 8) visible in the young adult female. The connection with the spermathecal duct is through the calyx, a cup-shaped structure that delimits a calyx cavity; within this cavity, the ventral side that is in contact with the calyx cavity, presents as a conspicuous cone-shaped structure (valve plug) in the middle of a helical slide-shaped surface that has two little holes (foramina). In the foramina there are small closing muscle fibres (Fig. 5f–g).

The spermathecal duct enlarges proximally, containing two small chambers separated by a central pierced wall (Figs. 5d-e,h). Distally the duct connects with the vagina (Fig. 3a,b; Supplementary Video S1).

The ovipositor (Figs. 1c, 2, 7, 8; Supplementary Video S3) is largely hidden inside the apical abdominal segments (Fig. 2a,c,d); only some apical parts can be observed externally (Fig. 1a-b,d). When segmented and isolated as a whole, three concentrically arranged valvulae (valves) are visible: 1^st^ (ventral), 2^nd^ (dorsal) and 3^rd^ (central) (Fig. 7, particularly 7a, 7g, 7h, 7j). The 1^st^ valvula is configured as a curved blade, forming a spoon-shaped structure that houses the 3^rd^ valvula and this in turn surrounds the 2^nd^ valvula both ventrally and laterally (Fig. 7g,j). The ovipositor protractor musculature is attached between the 1^st^ valvula and the inner side of the sub-genital plate (Figs. 1c, 8). Valvifer sclerites of the 1^st^ and 2^nd^ valvula are visible. The 1^st^ valvifer appears basally and it seems to enlarge acting as part of the wall of the basal genital chamber; the 2^nd^ valvifer appears as a dorsal broad sclerite extending from the base to 2/3 apical. The 2^nd^ valvula has, on each side, two longitudinal sclerotized reinforcement bars that join in a single lateral bar; from each side, these fuse medially in a pointed apical end (Fig. 7a,g–l)

Dorsally, a conspicuous longitudinal bar, the basal intervalvular sclerite (Fig. 7a,g,k), serves as an attachment for the ovipositor retractor musculature (Fig. 8a). The basal intervalvular sclerite articulates apically with three small vertical pointed plate sclerites: two lateral (latero-apical sclerites), two medial (medio-apical sclerites) and a single central one (centro-apical sclerite) (Fig. 7a,g-i, 8).

Basally at both sides of the ovipositor there are two vertical lateral plates that, dorsally, are curved forwards (Figs. 7a-g,i) with a conspicuous longitudinal apodemal ridge on the internal side (Fig. 7c–f).

The bacteriome organ (Figs. 3, 6; Supplementary Video S1) is situated dorsal to the ovaries and anterior to the spermatheca. It is well developed in the young adult female, extending along the anterior abdominal half (Fig. 3). The bacteriome is not visible in older adult females (Figs. 2, 8). It is roughly ‘H’ shaped, with two lateral lobes that have rounded ends. The two lateral lobes are united dorsally in the anterior third by a dorsal bridge which permits separation of each lateral lobe into a long anterior lobe and a short posterior lobe (Fig. 6a,c,e,g,h). The posterior side of the dorsal bridge, together with the posterior lobes, forms a concavity where the spermatheca fits. In a similar way, there is an anterior concavity in the anterior side of the dorsal bridge. The anterior lobes are anteriorly projected on each side of the digestive system. The filter chamber of the digestive system is located dorsally on the anterior concavity (Figs. 3, 6a,c,g,h; Supplementary Video S1). In section, the bacteriome shows an external layer of bacteriocytes, and an internal syncytium, containing symbiotic microorganisms, which at some points meets the surface of the bacteriome (Fig. 6b,d,f).

## Discussion

In psylloids the first abdominal segments are reduced, and attached to segments of the thorax or abdomen, so that it is difficult to interpret and number them. Thus, there has been no consensus concerning the visible sclerites of the abdominal segments^11,16,18,22,23^. In fact, publications on psylloids generally avoid the controversy and do not number these segments (e.g.^15,86^). In this study it was possible to clearly distinguish, dorsally, six tergite sclerites plus the proctiger in the abdomen of female *D. citri*. We interpret these as sclerites of the 3^rd^-7^th^ tergites (the 8^th^ was not visible because it was small and membranous), and the proctiger. Some authors consider that proctigers of psylloids are either the 9^th^ tergite^16^, a fusion of the 9^th^ and 10^th^ tergite^22,23^, or a fusion of the 9^th^-11^th^ tergites^11^. Ventrally, five sternal sclerites plus the subgenital plate were clearly visible. Moreover between the 7^th^ sternite and the subgenital plate two lateral sternites were apparent, which we interpreted as a divided 8^th^ sternite sclerite (Fig. 1d); this seems to be absent in other psylloid species^87^. The sub-genital plate of psylloids was described by Bitsch^11^ and other authors as the 9^th^ abdominal sternite^23,87^. However, Muir^16^ and Zucht^22^ working with *Psylla mali* and *P. crataegi*, respectively, interpreted it as the 7^th^ abdominal sternite, and numbered the visible abdominal sternites from 3–6 and 7 (sub-genital plate). Specifically, Muir^16^ wrote: “There is absolutely no evidence to support the idea that the sub-genital plate is formed by the ninth tergite, and there is no need to suppose that such an abnormal dislocation takes place”. As the problems of interpretation of the abdominal segment sclerites is not the main aim of this paper, we have adopted the numbering of the tergites and sternites that best fits with the general consensus, and particularly with Matsuda’s interpretation^23^ (Figs. 1–3).

In general the organization of the female reproductive system of *D. citri* that we observed agrees with previous descriptions for other psylloid species (e.g.^14,19,20,24,25,88,89^). Pesson^18^ stated that, in psylloids, each ovary has ca. 25 ovarioles. However, other reports have found up to 100 ovarioles^88,90^. In *D. citri* up to 50 ovarioles have been reported^24^ which is close to the number we counted, although it varied between ovaries: 40 in the right ovary and 46 in the left ovary.

In general the calyx (a dilatation of the oviduct for the reception of eggs) is located just after the conjunction of the ovarioles in insects^12,26,91,92^. However, the dilation we report here for *D. citri*, is configured as a dilation of the oviduct tube before it joins the common oviduct.

Various authors consider the female accessory glands to be synonymous with cement glands^12,26,91,92^. The role of the female accessory glands in insects is not well known. It has been suggested that their secretions may activate pheromone production^92^, or interact with proteins derived from the male accessory glands or his sperm^26^. In muscid flies, the accessory gland secretion contains proteolytic enzymes and an esterase that have essential roles in fertilization of the egg; they break down the acrosomal membrane of the sperm, and they lead to digestion of the cap over the micropyle. Whether this also occurs in other insects is not known^26^. *D. citri* has a pair of tubular, well developed, female accessory glands and, although their structure has been studied previously using electron microscopy, its function has not been elucidated^24^; currently their role is speculative and requires further research.

Protuberances of the spermathecal surface, which gave it a rough appearance, correspond to individual secretory cells of the epithelial secretor, as described previously for *Trioza alacris*^93^ and *D. citri*^24,25^. The spermatheca is not just a storage sac where spermatozoa are stored and/or nourished prior to egg fertilization. It is likely that the two materials with different densities that we observed in the spermatheca are not two separate cavities, but a single one containing a spermatodose. A spermatodose is a reorganization of the male spermatophore which had been previously transferred into the female spermatheca. The spermatodose protects the sperm from hostile spermiolytic activity (e.g.^93,94^). Thus, the ventral internal zone, appearing as high-density material, corresponds to the spermatodose. It is possible to distinguish denser filament structures within the spermatodose, which are of comparable size to spermatozoa as described previously for *D. citri*^95^, and similar to micro-CT-rendered images of spermatozoa inside testes^85^. The dorsal internal zone, appearing as low-density material, corresponds to nutritive liquid secretions of the epithelial secretor. In older adult females, which were close to ovipositing, the stored spermatodose in the internal cavity of the spermatheca had disaggregated to release spermatozoa; this explains the lack of two separate zones. The spermatheca appears to have all the impellent suction elements of a pump, which is as it has been described previously for the harlequin bug *Murgantia histrionica* by Stacconi & Romani^96^. In fact, the chambers and valve position in Stacconi & Romani’s Figs. 3a and 6 resemble the structures we observed in *D. citri* (Fig. 5d,e; Supplementary Video S2). The pumping function of the spermatheca has been reported in other insects as a spermathecal pump, consisting of muscle fibres located either in the spermathecal sac or in the duct wall. The presence of muscle fibres surrounding the entire spermathecal reservoir has been observed in a number of insect species (see revision by Pascini & Martins^94^). The presence of bundles of muscle fibrils in the spermathecal sheath of *D. citri* has also been described using transmission electron microscopy^24^. To pump the sperm into the spermatheca, the musculature of the spermathecal sheath contracts. It then relaxes and the elasticity of the spermathecal sheath returns the spermathecal sac to its globular shape, as the sperm are absorbed from the vagina through the spermathecal duct and pour into the calyx cavity. Once sperm reach the calyx cavity (the muscles of the foramina are relaxed so the orifices remain open), the calyx wall contracts so that the valve plug fits into the 1^st^ proximal chamber with its tip occluding the central hole in the wall that divides the two chambers (the hole is oblong-shaped to fit with the compressed shape of the valve plug). Thereafter the sperm glide through the two foramina into the lumen of the spermatheca, where they are protected within the spermatodose. When the female oviposits, the spermathecal pump procedure acts in reverse so that sperm are propelled into the spermatheca duct where the eggs are fertilized as they pass through the vagina at the point where the duct of the spermatheca connects.

The colleterial (= cement) gland produces a substance for attaching eggs to substrates, or for sticking them together in a mass as they are laid^12,26,88,92^. Some detailed studies of the structure of the colleterial gland have been published (e.g. in the lepidopteran *Sesamia nonagrioides*^97^). These glands are present in a number of Hemiptera and Homoptera^20^; its presence has been described in several different psylloid species (including *D. citri*^24^) as an unpaired globular structure connected, by a duct, to the distal third of the intervalvular space of the ovipositor^14,19,20,89^. The micro-CT showed a dense superficial layer corresponding to a secretory epithelium, and an internal cavity (lumen) filled with a less-dense material corresponding to the sticky secretions of the gland^24,89,97^. The colleterial gland duct pours its sticky secretion far further than the gonopore. Thus, once fertilized eggs enter the genital chamber and pass through the intervalvular space, they become coated with the sticky secretions of the colleterial gland just before they leave the ovipositor; this ensures they adhere to the citrus host plant.

For *P. mali*, Saunders described what he called a median accessory gland or parovarium^14,20^ which most likely corresponds to the bursa copulatrix described in a number of species in the Hemiptera and Homoptera^18^, including psylloid species (i.e.: *Trioza erytreae*^89^ and *Euphyllura phillyreae*^19^). However, both dissections of females^24^ and micro-CT confirm the absence of any bursa copulatrix in *D. citri*.

In many pterygote orders of insects, the female has an ovipositor that is highly evolved and well adapted for egg-laying and appears to be formed of the appendages of the 8^th^ and 9^th^ abdominal segments^12.^ In Hemiptera the ovipositor has rather a complex structure^12,18,98,99^. Indeed, the lateral view of the female terminalia, with the lateral outline view of the ovipositor and ventral valvular, have been used as an additional taxonomic character for identification of female psylloids (e.g.^17,86,100–102^). Despite this, Hodkinson & White stated that ovipositor structures were generally of little taxonomic value^15^. The structure of the ovipositor has been described in detail for different psylloid species (e.g. *P. mali*^14,16,20^ *P. crataegi*^11,22^, *Pachypsylla celtidis*^10^). Moreover, published light microscopy images from slide preparations show partial views of the distal 2/3^rds^ of the ovipositor of *E. phillyreae*^19^ and *D. citri*^24^.

Interpretation of the elements of the ovipositor vary depending on author. Most recognize and number the valvulae, but there are differences in the interpretation of individual structures. Thus, Muir^16^ described the lateral plate sclerites as apodemes of the 8^th^ abdominal sternites, and called them lateral plates (a term that we maintain here); later, Zucht^22^ considered them as the 1^st^ valvifers. Recently Austin^10^ considered each lateral plate to represent a gonangulum (the sclerite that acts as a fulcrum, articulating against the 1^st^ valvifer, and coupled with the 2^nd^ and 3^rd^ valvulae), but Matsuda^23^ interpreted the lateral plates as a fusion of the 1^st^ valvifer and the gonangulum. Homology amongst gonangula from different insect groups remains unclear^103^. We maintain the general term ‘lateral plates’, as used by Muir^16^, even though they may represent the gonangulum as Austin^10^ suggested for *P. celtidis* (Psyllidae). To the best of our knowledge there is no detailed study of the ovipositor in *D. citri*. Therefore, what we present in this paper represents not only the first detailed study of this structure in *D. citri*, but also the most complete detailed view of the ovipositor of any psylloid species using new micro-CT techniques.

The micro-CT-rendered images and 3D reconstructions of the bacteriome that we obtained are completely comparable to, and clearer than, those previously published for the leafhopper *O. albicinctus* which had been obtained using a synchrotron^33^. Moreover, the detailed structure is comparable to confocal micrographs of histological bacteriome sections made using fluorescence *in situ* hybridization (FISH)^29–31,33,35,36^. In fact, confocal FISH micrographs (Fig. 1a,d of Dan *et al*.,^35^) of the *D. citri* bacteriome look exactly the same as our micro-CT-rendered images (Fig. 6d,f). These images showed an external layer of bacteriocytes, harbouring *Candidatus* Carsonella ruddii (β-Proteobacteria), and an internal syncytium, harbouring *Candidatus* Profftella armatura (γ-Proteobacteria)^31,35,36^, which extended to the surface of the bacteriome, and was also clearly visible with micro-CT. Recent evidence has reported the presence of *Wolbachia*, another endosymbiotic bacteria, in the *D. citri* bacteriome^36^.

## Methods

The six older mated adult female *D. citri* specimens for this study came from the rearing facilities at the United States Department of Agriculture, Agriculture Research Service, Fort Pierce, Florida (USA). These specimens (shown in Figs. 1, 2, 7, 8; Supplementary Video S3) were fed for three days on an orange tree sprig submerged in BAPC (Branched Amphiphilic Peptide Capsules) linked to Hg as a contrast agent^104^. They were rinsed three times in 30% ethanol (10 min for each rinse), dehydrated in an ethanol series (30 min per step, 50%, 70%, 80%, 90%, 95%, and three times at 100%), chemically dried by submersion in 2 ml of 100% hexamethyldisilazane (HMDS) for 2 hours and dried overnight at 35 °C. In addition, a young mated adult female from the facilities at the University of Florida, Department of Entomology and Nematology was also processed (Figs. 3–5; Supplementary Videos S1 and S2). It was provided by Dr Joseph M. Cicero who separated the abdomen and removed the apical part to facilitate the entry of chemicals during preparation (fixed with 4% formaldehyde and 1.5% glutaraldehyde, rinsed and then incubated in 1% OsO4 for 20 minutes, then rinsed again and dehydrated in an ethanol series, and critical point dried).

All specimens were glued with cyanoacrylate to the tip of a nylon fishing line 200 µm in diameter, as previously described^105,106^. The prepared specimens were scanned using a SkyScan 1172 desktop high-resolution micro-CT, with a Hamamatsu L702 source and a Ximea 11Mp camera. The following setting parameters were used: (a) for the older adult females: isotropic voxel size = 0.52 µm per pixel; source voltage = 48KV, source current = 68 µA, image rotation step = 0.2°, 360° rotation scan and no filter, resulting in 1802 ×-ray images; (b) for the young adult female: isotropic voxel size = 0.99 µm per pixel; source voltage = 49KV, source current = 51 µA, image rotation step = 0.3°, 360° rotation scan and no filter, resulting in 1202 ×-ray images.

The most recent versions of the Bruker micro-CT’s Skyscan software (NRecon v.1.7.4.6, DataViewer v.1.5.6.2, CTAnalyser v.1.18.8.0, https://www.bruker.com/products/microtomography.html) were used for primary reconstructions and the ‘cleaning’ process to obtain datasets on ‘slices’ through the specimens, as described previously^105^. Amira’s software, v. 6.7.0 (Thermo Fisher Scientific, Waltham, MA)^107,108^ (with the built-in ‘volrenRed.col’ colour filter) was used to obtain volume-rendered images (Figs. 1–7; Supplementary Videos S1–S3). Different anatomical parts were independently segmented to obtain the final rendered images used in Figs. 2–7. After segmentation, each structure was subjected to an arithmetic operation to obtain the actual texture of structures in desired colours. Specifically: A*(B > 0), where A represents the whole specimen and B the segmented structure. The Amira’s software, multiplanar slice view was used to obtain the rendered images in Fig. 8.

In accordance with the micro-CT results (as seen in the Figs.), standard anatomical positions are used to describe structures. Proximal/distal anatomical terminology for any duct departing from an organ is named according to its proximal or distal position in relation to the organ itself (as for instance in Fig. 1 of Slater^109^).

For consistency, and to avoid poor or misleading descriptions of any structure or form as a result of undesired deformation, the structures visualized and described in this study were checked and found to exist and maintain their shape and position in each of the seven females that were scanned and reconstructed.

## Supplementary information

Supplementary information 1.

Supplementary information 2.

Supplementary information 3.

## Data Availability

The datasets generated and analyzed during the course of the study are available from J.A.T/I.A.A. upon reasonable request.
